# Older Adults’ Continued Use of Smart Healthcare Applications: An Integrated HBM–UTAUT Model with Technology Anxiety

**DOI:** 10.3390/healthcare14172790

**Published:** 2026-09-01

**Authors:** Yunho Ji, Joonho Moon

**Affiliations:** College of Business Administration, Kangwon National University, Chuncheon 24341, Republic of Korea; yunho.ji@kangwon.ac.kr

**Keywords:** smart healthcare applications, older adults, UTAUT, health belief model, technology anxiety, use behavior

## Abstract

Background/Objectives: This study examines the continued use of smart healthcare applications among older adults by integrating the Health Belief Model (HBM) with the Unified Theory of Acceptance and Use of Technology (UTAUT). Perceived health threat and health management self-efficacy were considered health-related antecedents, while technology anxiety was examined as a moderator. Methods: Data were collected from 224 South Korean adults aged 60 years or older with prior experience using smart healthcare applications. Confirmatory factor analysis and structural equation modeling were used to assess the measurement and structural models. Indirect effects were tested using 5000 bootstrap resamples, and moderation was examined through hierarchical regression and simple slope analysis. Results: Perceived health threat was positively associated with performance expectancy but not with effort expectancy. Health management self-efficacy was positively associated with both performance and effort expectancy. Performance expectancy, effort expectancy, social influence, and facilitating conditions were all positively associated with behavioral intention, which showed the strongest relationship with actual use behavior. Technology anxiety significantly weakened the relationship between social influence and behavioral intention, whereas its moderating effects on performance expectancy and effort expectancy were not significant. Conclusions: The findings provide an integrated explanation of continued smart healthcare application use among older adults by linking health-related beliefs, UTAUT factors, and technology anxiety. Sustained use may be promoted by strengthening self-efficacy, improving usability, and reducing technology-related anxiety through accessible design and appropriate support.

## 1. Introduction

Population aging and digital transformation are simultaneously reshaping healthcare among older adults, and South Korea provides a particularly relevant context in which to examine this transition. In 2025, adults aged 65 years or older accounted for 20.3% of the Korean population, and this proportion is projected to exceed 30% by 2036 and 40% by 2050 [1]. At the same time, digital engagement among Korean older adults has increased substantially; in 2024, 76.9% of adults aged 65 years or older used the Internet [1]. These demographic and digital transitions create both a growing need and an expanding opportunity for technology-supported self-care among older adults. Smart healthcare applications support continuous monitoring and management of health indicators such as physical activity, blood pressure, blood glucose, medication adherence, and sleep patterns [2,3,4]. These applications can facilitate a shift from treatment-centered healthcare toward preventive and self-management approaches, which is particularly relevant for older adults who often require continuous health management [5,6]. However, their benefits depend on older adults’ willingness to adopt and continuously use them [7]. Older adults may face distinctive barriers to technology adoption, including limited digital experience, usability difficulties, and psychological barriers associated with technology use [8,9,10].

The Unified Theory of Acceptance and Use of Technology (UTAUT) provides a well-established framework for explaining technology adoption through performance expectancy, effort expectancy, social influence, and facilitating conditions [11]. Although UTAUT has been widely applied to digital health technologies, relying primarily on technology-related determinants may not fully explain the adoption of smart healthcare applications among older adults. Unlike general information technologies, these applications are used specifically for health monitoring, disease prevention, and self-management [12,13]. Thus, older adults’ evaluations of such technologies may depend not only on expected technological benefits and ease of use but also on their perceptions of health risks and confidence in managing their own health [14,15].

This suggests an important research gap. Previous studies have provided substantial evidence regarding technological determinants of digital health adoption, but relatively less attention has been paid to how health-related beliefs operate as antecedents of technology acceptance expectations among older adults. The Health Belief Model (HBM) offers a useful theoretical basis for addressing this gap because perceived health threat and self-efficacy capture individuals’ motivation to engage in health-related behaviors. Integrating these health-related factors with UTAUT therefore allows health motivations to be linked to evaluations of technological usefulness and ease of use. In addition, although technology anxiety has been recognized as an important psychological barrier to technology adoption among older adults, its role in altering the strength of the relationships between UTAUT determinants and behavioral intention remains insufficiently understood. Examining technology anxiety as a moderator, rather than merely as an additional predictor, may therefore reveal when positive evaluations and social encouragement are less likely to translate into behavioral intention.

Accordingly, this study integrates HBM-related health factors with UTAUT by positioning perceived health threat and health management self-efficacy as antecedents of performance expectancy and effort expectancy and by examining technology anxiety as a moderator of the relationships between key UTAUT factors and behavioral intention. The study further examines whether behavioral intention translates into actual use behavior. In doing so, this study extends conventional technology acceptance research in two ways: first, by linking health-related beliefs to the formation of technology acceptance expectations; and second, by identifying technology anxiety as a psychological boundary condition in the acceptance process. This integrated perspective provides a more context-sensitive explanation of smart healthcare application adoption among older adults and offers implications for the design and implementation of age-friendly digital healthcare services.

## 2. Theoretical Background

### 2.1. Smart Healthcare Applications and Technology Adoption Among Older Adults

Smart healthcare applications are digital tools that utilize mobile devices and information and communication technologies (ICT) to collect and manage individuals’ health information while supporting health monitoring and health-promoting behaviors [7,16]. These applications provide various functions, including tracking physical activity, sleep, heart rate, blood pressure, blood glucose, medication adherence, personalized health information, and remote healthcare services [17,18]. By enabling users to continuously monitor and manage their health in everyday life, smart healthcare applications have become an important means of promoting preventive and self-directed health management [2,3]. More broadly, digital health technologies have considerable potential to enhance functional independence, support healthy aging, and improve the quality of life of older adults [19,20].

Smart healthcare applications may be particularly useful for older adults because many require ongoing support for chronic health conditions [21]. However, older adults are a heterogeneous population, and their use of digital health technologies may vary according to prior digital experience, education, socioeconomic resources, health and functional status, and access to support services. Thus, age alone should not be assumed to determine digital health capacity. When appropriately matched to individual needs, smart healthcare applications can support health monitoring, self-care, and communication with healthcare professionals. Previous studies have shown that digital health interventions can improve physical activity, self-management, and health outcomes among older adults [22,23].

Despite these potential benefits, the successful implementation of smart healthcare applications depends largely on older adults’ willingness to adopt and continuously use the technology [24]. Barriers to use may vary considerably among older adults depending on their physical and cognitive conditions, digital experience, access to support, and concerns about privacy and data security [25]. In addition, small font sizes, complex menus, burdensome authentication, and poorly organized interfaces may create usability difficulties for some users [26]. Therefore, smart healthcare applications should accommodate diverse user capabilities through accessible interfaces, appropriate technical support, and adequate privacy safeguards [10,27].

Privacy and health data protection are particularly important because smart healthcare applications collect and process sensitive personal health information. Previous studies have shown that privacy concerns can reduce older adults’ willingness to use digital health services, while transparent data practices and greater control over health information may facilitate acceptance [28,29,30].

Technology adoption among older adults is influenced not only by perceived usefulness and ease of use but also by social support, health status, digital competence, and psychological factors [8,9,14]. Given the heterogeneity of older adults in health conditions and digital experience [31], their technology use should not be understood solely in terms of age-related vulnerability. Because all participants had prior experience using smart healthcare applications, this study focuses on post-adoption acceptance and continued use rather than initial adoption. Accordingly, UTAUT is extended with health-related antecedents and technology anxiety to explain behavioral intention and actual use behavior.

### 2.2. Health-Related Factors and Technology Acceptance Expectations

Smart healthcare applications are purpose-driven technologies that are directly associated with individuals’ perceptions of health risks and motivation for health-related behaviors. Therefore, explaining older adults’ adoption of smart healthcare applications requires consideration not only of the technological characteristics of the applications but also of how individuals perceive their health risks and the extent to which they believe they can effectively manage [32]. Previous studies have shown that technology adoption among older adults is influenced not only by technological and social factors but also by individual characteristics, including health status, psychological traits, and self-efficacy [33,34].

In the present study, perceived health threat and health management self-efficacy are proposed as antecedents of performance expectancy and effort expectancy [15,35,36]. Performance expectancy refers to the belief that using smart healthcare applications will enhance health management outcomes, whereas effort expectancy represents the perceived ease of learning and using these applications [37,38]. Rather than assuming that health-related factors directly influence behavioral intention, this study posits that they shape individuals’ evaluations of the usefulness and usability of smart healthcare applications, which subsequently affect the technology adoption process. This perspective integrates health behavior theory with technology acceptance theory, providing a theoretical explanation of how older adults develop perceptions of smart healthcare applications as useful and easy-to-use technologies for health management.

#### 2.2.1. Perceived Health Threat, Performance Expectancy, and Effort Expectancy

Perceived health threat refers to individuals’ perceptions of their susceptibility to health problems and the seriousness of their potential consequences. In the Health Belief Model (HBM), it comprises perceived susceptibility and perceived severity and represents a key cognitive determinant of preventive health behavior [37,38]. Individuals perceiving greater health threats are more likely to seek preventive actions and effective health-management strategies [39]. Extending this perspective, the present study positions perceived health threat as a health-related antecedent of UTAUT expectations, thereby linking health motivation to technology acceptance rather than treating the two processes separately. This integration provides a theoretical basis for examining whether perceived health threat is associated with older adults’ performance expectancy and effort expectancy regarding smart healthcare applications [40,41].

Older adults who perceive themselves to be at greater risk of health deterioration or chronic diseases are more likely to view smart healthcare applications as useful tools for monitoring health conditions, managing health behaviors, and preventing disease progression [35,42]. As the perceived need for health outcomes increases, individuals are expected to place greater value on the health benefits and performance outcomes that can be achieved through the use of these applications. Accordingly, perceived health threat is expected to positively influence the formation of performance expectancy.

Furthermore, older adults with higher perceived health threat may be more motivated to learn and utilize new technologies for health management. The perceived necessity of addressing health problems may lead individuals to regard the time and effort required to learn and use smart healthcare applications as worthwhile, thereby enhancing their expectations regarding the ease of adopting the technology [43,44]. However, perceived health threat itself does not directly alter the objective usability of the technology. Rather, its influence on effort expectancy should be understood as reflecting a motivational evaluation of the willingness to invest effort in learning and using the application [45,46]. Based on these arguments, the following hypotheses are proposed:

**H1-1.**  *Perceived health threat positively influences performance expectancy.*

**H1-2.**  *Perceived health threat positively influences effort expectancy.*

#### 2.2.2. Health Management Self-Efficacy, Performance Expectancy, and Effort Expectancy

Health management self-efficacy refers to an individual’s belief in their capability to plan and perform the behaviors necessary to maintain or improve their health [47]. Individuals with high self-efficacy are more likely to actively engage in health-promoting behaviors and persist in achieving their health goals despite obstacles or challenges. In the health behavior literature, self-efficacy has consistently been identified as a key predictor of preventive health behaviors and effective health management [37,48].

Older adults with higher health management self-efficacy are more likely to perceive smart healthcare applications as practical and valuable tools for supporting their health management [49]. Confidence in one’s ability to record health information, monitor health status, and follow the recommendations provided by these applications is expected to enhance beliefs regarding the performance benefits of using the technology [50]. Consequently, health management self-efficacy is expected to strengthen performance expectancy by increasing older adults’ perceptions of the perceived health benefits of smart healthcare applications.

Health management self-efficacy is also closely associated with individuals’ evaluations of the effort required to use new technologies. Older adults who are confident in their ability to manage their own health are more likely to believe that they can successfully learn and utilize the functions of smart healthcare applications, even when the learning process involves certain challenges [51]. Conversely, individuals with lower self-efficacy may perceive the same applications as more complicated and difficult to use. Previous studies on mobile health (mHealth) adoption among older adults have similarly identified self-efficacy as an important antecedent of both performance expectancy and effort expectancy [52,53]. Based on these arguments, the following hypotheses are proposed:

**H2-1.**  *Health management self-efficacy positively influences performance expectancy.*

**H2-2.**  *Health management self-efficacy positively influences effort expectancy.*

### 2.3. UTAUT Factors, Behavioral Intention, and Use Behavior

The Unified Theory of Acceptance and Use of Technology (UTAUT) offers a consolidated framework for understanding why individuals intend to use and continue using technology. Developed by Venkatesh et al. [11], the model synthesizes major perspectives from earlier technology acceptance theories, including TAM, TRA, TPB, and IDT. Rather than viewing technology use as the outcome of a single perception, UTAUT explains acceptance through several complementary considerations: expected benefits, perceived effort, social influence, and the availability of supportive conditions. These dimensions are represented by performance expectancy, effort expectancy, social influence, and facilitating conditions. Within the framework, performance expectancy, effort expectancy, and social influence are primarily associated with behavioral intention, whereas facilitating conditions and behavioral intention are linked to actual technology use.

#### 2.3.1. UTAUT and Behavioral Intention

The UTAUT framework is particularly suitable for explaining older adults’ adoption of smart healthcare applications because it considers not only individuals’ cognitive evaluations of technology but also social influences and environmental conditions that facilitate technology use [15,36]. Older adults’ acceptance of smart healthcare applications is likely to be shaped by their expectations regarding the health benefits of the applications, perceptions of the effort required to learn and use them, recommendations from family members, friends, and healthcare professionals, and the availability of devices, knowledge, training, and technical support [54,55]. Because smart healthcare applications require users to record health information, monitor physiological indicators, and engage in continuous self-management, their adoption cannot be adequately explained solely by the functional benefits of the technology. Therefore, UTAUT provides a comprehensive theoretical framework for understanding how older adults develop behavioral intentions to adopt smart healthcare applications.

Performance expectancy refers to the degree to which individuals believe that using smart healthcare applications will improve their health management outcomes [56]. Older adults who perceive that these applications can effectively support health monitoring, disease prevention, and health improvement are expected to demonstrate stronger intentions to adopt the technology [57,58]. Because smart healthcare applications are designed primarily to facilitate continuous health monitoring and self-management, expected health-related benefits are likely to constitute an important determinant of behavioral intention.

Effort expectancy refers to the degree to which individuals perceive smart healthcare applications as easy to learn and use. For older adults, complicated menu structures, complex authentication procedures, and excessive data entry requirements may discourage technology adoption [59]. Consequently, perceived ease of use becomes an important criterion in determining behavioral intention. When older adults perceive that an application is simple to operate and that its functions are easy to understand, the perceived burden of technology use decreases, thereby strengthening their intention to use the application.

Social influence refers to the extent to which individuals perceive that important others, such as family members, friends, and healthcare professionals, believe they should use smart healthcare applications [60]. Older adults often rely on the experiences and recommendations of significant others when evaluating the usefulness and trustworthiness of unfamiliar technologies [34]. Recommendations from family members, endorsements by healthcare professionals, and positive experiences shared by peers may reduce uncertainty regarding technology use and consequently strengthen behavioral intention [61,62].

Facilitating conditions refer to individuals’ perceptions of the availability of the resources and support necessary for using smart healthcare applications, including appropriate devices, knowledge, training, and technical assistance. Older adults are more likely to adopt new technologies when adequate support systems are available to help them overcome difficulties encountered during technology use [63,64]. Accordingly, assistance from family members or professionals, user education, and accessible support services are expected to reduce the practical barriers associated with technology use and strengthen behavioral intention.

Based on the above arguments, the following hypotheses are proposed:

**H3-1.**  *Performance expectancy positively influences behavioral intention to use smart healthcare applications.*

**H3-2.**  *Effort expectancy positively influences behavioral intention to use smart healthcare applications.*

**H3-3.**  *Social influence positively influences behavioral intention to use smart healthcare applications.*

**H3-4.**  *Facilitating conditions positively influence behavioral intention to use smart healthcare applications.*

#### 2.3.2. Behavioral Intention and Use Behavior

Behavioral intention refers to an individual’s willingness and plan to use a particular technology in the future and is widely regarded as the most immediate antecedent of actual technology use. Older adults with stronger behavioral intentions to use smart healthcare applications are more likely to install the applications, enter and monitor health-related information, and actively utilize their functions for health management [65,66].

However, among older adults, a positive behavioral intention does not always translate into actual use. Practical barriers, such as application installation, user registration, device connectivity, and troubleshooting, may hinder the transition from intention to behavior [67]. Nevertheless, a strong behavioral intention provides the motivational foundation that enables individuals to overcome these obstacles and initiate or continue technology use [68]. Consistent with the UTAUT framework, behavioral intention is therefore expected to positively influence actual use behavior. Accordingly, the following hypothesis is proposed:

**H4.**  *Behavioral intention to use smart healthcare applications positively influences use behavior.*

### 2.4. The Moderating Role of Technology Anxiety

Technology anxiety refers to feelings of tension, apprehension, fear of errors, and loss of control when using digital technologies [69]. Previous studies have shown that technology anxiety can directly reduce technology acceptance among older adults by increasing psychological discomfort and reluctance toward technology use [70,71]. However, technology anxiety may also shape how individuals respond to perceived usefulness, ease of use, and social encouragement. Thus, its role may extend beyond a direct predictor to influence the strength of relationships between established technology acceptance factors and behavioral intention.

Accordingly, this study focuses on technology anxiety as a moderator rather than an additional direct predictor. This approach does not exclude its potential direct association with technology acceptance; rather, it aims to examine whether different levels of technology anxiety alter how key UTAUT determinants are associated with behavioral intention. In this sense, technology anxiety is conceptualized as a psychological boundary condition in the technology acceptance process. Older adults with low technology anxiety may more readily translate positive evaluations of usefulness and ease of use, as well as social encouragement, into behavioral intention. Conversely, when technology anxiety is high, these positive relationships may become weaker because concerns about technology use can limit the extent to which favorable evaluations and external encouragement are associated with behavioral intention [70,72,73].

In contrast, older adults with high levels of technology anxiety may remain reluctant to adopt smart healthcare applications even when they perceive the technology as useful and easy to use. Concerns about operational errors, difficulties in using digital devices, and the potential loss of control during technology use may weaken the positive influence of performance expectancy and effort expectancy on behavioral intention [68,70]. Likewise, recommendations from significant others may not be sufficient to overcome the psychological barriers associated with technology anxiety. Therefore, technology anxiety is expected to function as a boundary condition that weakens the positive effects of performance expectancy, effort expectancy, and social influence on behavioral intention toward smart healthcare applications. Based on these arguments, the following hypotheses are proposed:

**H5-1.**  *Technology anxiety negatively moderates the relationship between performance expectancy and behavioral intention, such that the positive effect of performance expectancy on behavioral intention becomes weaker as technology anxiety increases.*

**H5-2.**  *Technology anxiety negatively moderates the relationship between effort expectancy and behavioral intention, such that the positive effect of effort expectancy on behavioral intention becomes weaker as technology anxiety increases.*

**H5-3.**  *Technology anxiety negatively moderates the relationship between social influence and behavioral intention, such that the positive effect of social influence on behavioral intention becomes weaker as technology anxiety increases.*

## 3. Research Method

### 3.1. Research Model and Hypotheses

The proposed research model was grounded in the Unified Theory of Acceptance and Use of Technology (UTAUT) to examine older adults’ behavioral intention and continued use of smart healthcare applications. In line with the UTAUT framework, performance expectancy, effort expectancy, social influence, and facilitating conditions were positioned as key antecedents of behavioral intention. Behavioral intention, in turn, was hypothesized to be positively associated with actual use behavior.

Considering that smart healthcare applications differ from general information technologies in that they are specifically designed to support health management, this study further incorporated perceived health threat and health management self-efficacy as antecedents of performance expectancy and effort expectancy. Perceived health threat refers to individuals’ perceptions of the likelihood and seriousness of potential health problems, whereas health management self-efficacy refers to individuals’ confidence in their ability to perform behaviors necessary to maintain or improve their health. It was assumed that these health-related factors shape older adults’ evaluations of the expected usefulness and ease of use of smart healthcare applications.

**Figure 1:**
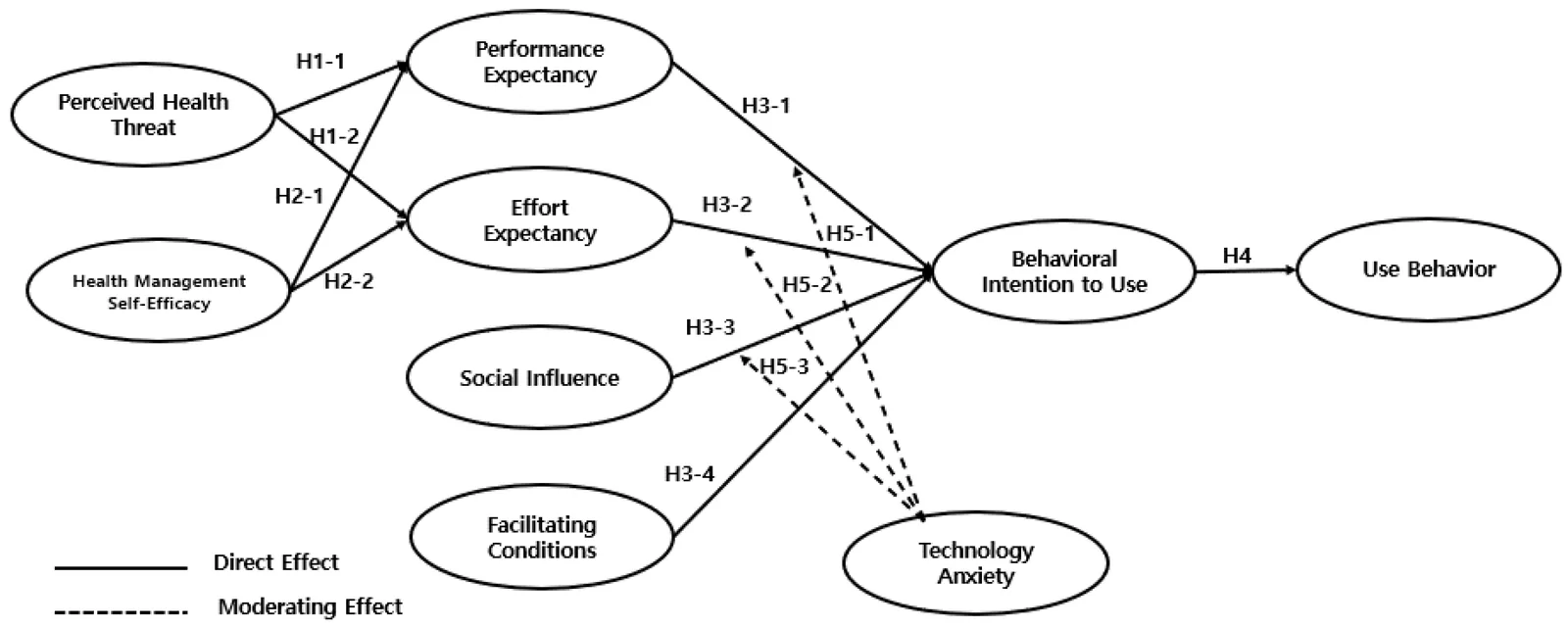
The proposed research model and the corresponding hypotheses.

In addition, technology anxiety was incorporated as a moderating variable. Technology anxiety was defined as the feelings of tension, apprehension, and concerns about making errors that older adults may experience when using digital technologies. It was hypothesized to weaken the positive effects of performance expectancy, effort expectancy, and social influence on behavioral intention. Accordingly, the proposed research model consists of health-related antecedents, the core UTAUT constructs, behavioral intention, use behavior, and the moderating role of technology anxiety. The proposed research model and the corresponding hypotheses are presented in Figure 1.

### 3.2. Measurement Instruments

The measurement instruments were adapted from previously validated scales to reflect the context of smart healthcare application use among older adults. The study included nine latent constructs: perceived health threat, health management self-efficacy, performance expectancy, effort expectancy, social influence, facilitating conditions, technology anxiety, behavioral intention, and use behavior. All items were measured on a five-point Likert scale ranging from 1 (strongly disagree) to 5 (strongly agree), with higher scores indicating higher levels of the corresponding construct.

Performance expectancy, effort expectancy, social influence, facilitating conditions, behavioral intention, and use behavior were adapted from the UTAUT measures developed by Venkatesh et al. [11]. Performance expectancy assessed the perceived benefits of smart healthcare applications for self-management, while effort expectancy reflected perceived ease of learning and use. Social influence measured encouragement from significant others, and facilitating conditions represented the availability of resources and support for application use. Behavioral intention reflected willingness to continue using smart healthcare applications, whereas use behavior represented their actual utilization for health management. The original items were modified to fit the smart healthcare context while retaining their conceptual meanings.

Based on the Health Belief Model, perceived health threat represented individuals’ perceptions of their susceptibility to health problems and the seriousness of their consequences. Health management self-efficacy reflected confidence in planning, performing, and sustaining behaviors necessary to maintain or improve health, drawing on previous research identifying self-efficacy as an important factor in digital health adoption among older adults [74].

Technology anxiety was adapted from Meuter et al. [75] and defined as negative emotional responses to digital technology, including tension, apprehension, fear of making errors, and perceived loss of control. Higher scores therefore indicated greater anxiety about using digital technologies rather than lower technological competence.

Finally, because use behavior was assessed in terms of the frequency and extent of application use for health management, the sample was restricted to older adults with prior experience using smart healthcare applications. Respondents were instructed to answer the questions with a specific application in mind, thereby reducing conceptual ambiguity in the self-reported assessment of actual use behavior.

### 3.3. Data Collection, Sample, and Data Analysis

Participants were recruited through convenience sampling with the assistance of undergraduate and graduate students, who invited relatives and acquaintances aged 60 years or older who met the eligibility criteria. Efforts were made to include participants with diverse demographic characteristics; however, this approach may have favored older adults with greater digital competence or functional capacity. Prior to the main survey, a pilot test was conducted to assess item clarity and comprehensibility, and minor revisions were made accordingly. An a priori power analysis using G*Power 3.1.9.7. indicated a minimum sample of 85 participants, assuming a medium effect size (f2 = 0.15), α = 0.05, power = 0.80, and four predictors. After data screening, 224 valid questionnaires were retained. Common method bias was assessed using Harman’s single-factor test, with the first factor accounting for 20.80% of the total variance, below the 50% threshold, suggesting that common method bias was not a serious concern.

This study was conducted in accordance with established ethical principles for human-subject research, including respect for persons, beneficence, and justice. Before participating in the survey, all respondents were informed of the purpose of the study, the survey procedures, the intended use of the collected data, the voluntary nature of participation, the protection of anonymity, and their right to withdraw from the study at any time without penalty. Only individuals who voluntarily agreed to participate were included in the survey. In accordance with the Bioethics and Safety Act of the Republic of Korea, no personally identifiable information, such as names, telephone numbers, or residential addresses, was collected. All survey responses were anonymized prior to analysis, and the data were used exclusively for research purposes. The findings are reported only in aggregate form to ensure that no individual participant can be identified.

All statistical analyses were conducted using IBM SPSS Statistics 27.0 and IBM SPSS AMOS 27.0 (IBM Corp., Armonk, NY, USA). SPSS 27.0 was used for descriptive statistics, correlation, reliability, and moderation analyses, whereas AMOS 27.0 was used for confirmatory factor analysis (CFA), structural equation modeling (SEM), and bootstrapping.

## 4. Results

### 4.1. Respondent Characteristics

As presented in Table 1, a total of 224 valid responses were included in the final analysis. Female respondents accounted for 56.3% of the sample, while males represented 43.8%. The largest age group was 60–64 years (45.1%), followed by 65–69 years (35.3%) and 70 years or older (19.6%). Regarding self-rated health status, the largest proportion of respondents reported their health as fair (44.2%), followed by poor (33.5%). In terms of smart healthcare application use, 27.7% of the respondents had used such applications for 1–3 years, and 46.0% reported using them two to four times per week. Regarding the primary type of application used, Fitness & Activity was the most frequently reported category (30.8%), followed by Diet & Nutrition (25.9%), Sleep & Mental Health (14.3%), Medical & Chronic Disease Management (11.6%), Community & Gamification (9.4%), and Medical Information & Telehealth (8.0%).

### 4.2. Measurement Model Assessment

Before testing the structural model, a confirmatory factor analysis (CFA) was conducted to assess the reliability and validity of the measurement model. As shown in Table 2, the measurement model included nine latent constructs: perceived health threat, health management self-efficacy, performance expectancy, effort expectancy, social influence, facilitating conditions, technology anxiety, behavioral intention, and use behavior. One measurement item for social influence (SI4) was removed during the validation process because it did not satisfy the recommended measurement criteria.

The measurement model demonstrated an acceptable fit to the data (χ^2^ = 486.414, df = 459, *p* = 0.182, χ^2^/df = 1.060, CFI = 0.991, TLI = 0.989, RMSEA = 0.016, SRMR = 0.046). The χ^2^/df value was well below the recommended threshold of 3.0, while the CFI and TLI values exceeded 0.90. In addition, both RMSEA and SRMR were below the recommended cutoff value of 0.08, indicating that the overall measurement model exhibited a satisfactory fit [76,77,78].

The standardized factor loadings ranged from 0.657 to 0.836, exceeding the recommended minimum value of 0.50, indicating that all retained indicators adequately represented their corresponding latent constructs. Furthermore, Cronbach’s α values ranged from 0.790 to 0.848, and composite reliability (CR) values ranged from 0.792 to 0.850, both exceeding the recommended threshold of 0.70. The average variance extracted (AVE) ranged from 0.543 to 0.635, surpassing the recommended criterion of 0.50. These results indicate that the measurement model demonstrated satisfactory internal consistency, convergent validity, and construct reliability.

### 4.3. Correlation Analysis and Discriminant Validity

To examine the relationships among the constructs and assess discriminant validity, Pearson correlation analysis was conducted. In addition, following the criterion proposed by Fornell and Larcker [79], discriminant validity was evaluated by comparing the square root of the average variance extracted (AVE) for each latent construct with the corresponding inter-construct correlation coefficients, as presented in Table 3.

The results indicated that the Pearson correlation coefficients among the constructs ranged from −0.157 to 0.490. Overall, the correlations were generally consistent with the hypothesized relationships. Technology anxiety exhibited significant negative correlations with effort expectancy (r = −0.148, *p* < 0.05) and facilitating conditions (r = −0.157, *p* < 0.05). In contrast, behavioral intention was positively correlated with performance expectancy (r = 0.359, *p* < 0.001), effort expectancy (r = 0.470, *p* < 0.001), social influence (r = 0.284, *p* < 0.001), and facilitating conditions (r = 0.314, *p* < 0.001). Furthermore, behavioral intention showed a significant positive correlation with use behavior (r = 0.418, *p* < 0.001).

According to the Fornell–Larcker criterion, the square roots of the AVE values ranged from 0.737 to 0.797, and each exceeded the corresponding inter-construct correlations. The highest correlation was observed between health management self-efficacy and effort expectancy (r = 0.490), which remained lower than the square roots of their respective AVE values (0.766 and 0.753). Similarly, the correlation between behavioral intention and use behavior (r = 0.418) was lower than the square roots of the AVE values for the two constructs (0.797 and 0.748). These findings provide evidence of satisfactory discriminant validity for the measurement model.

### 4.4. Structural Model Assessment

The structural model demonstrated an acceptable fit to the data (Table 4). The chi-square statistic was 415.052 with 367 degrees of freedom, resulting in a χ^2^/df ratio of 1.131, which was well below the recommended threshold of 3.0. Although the chi-square test was statistically significant (*p* = 0.042), the chi-square statistic is known to be sensitive to sample size and model complexity. Therefore, additional model fit indices were considered to evaluate the overall adequacy of the structural model.

**Figure 2:**
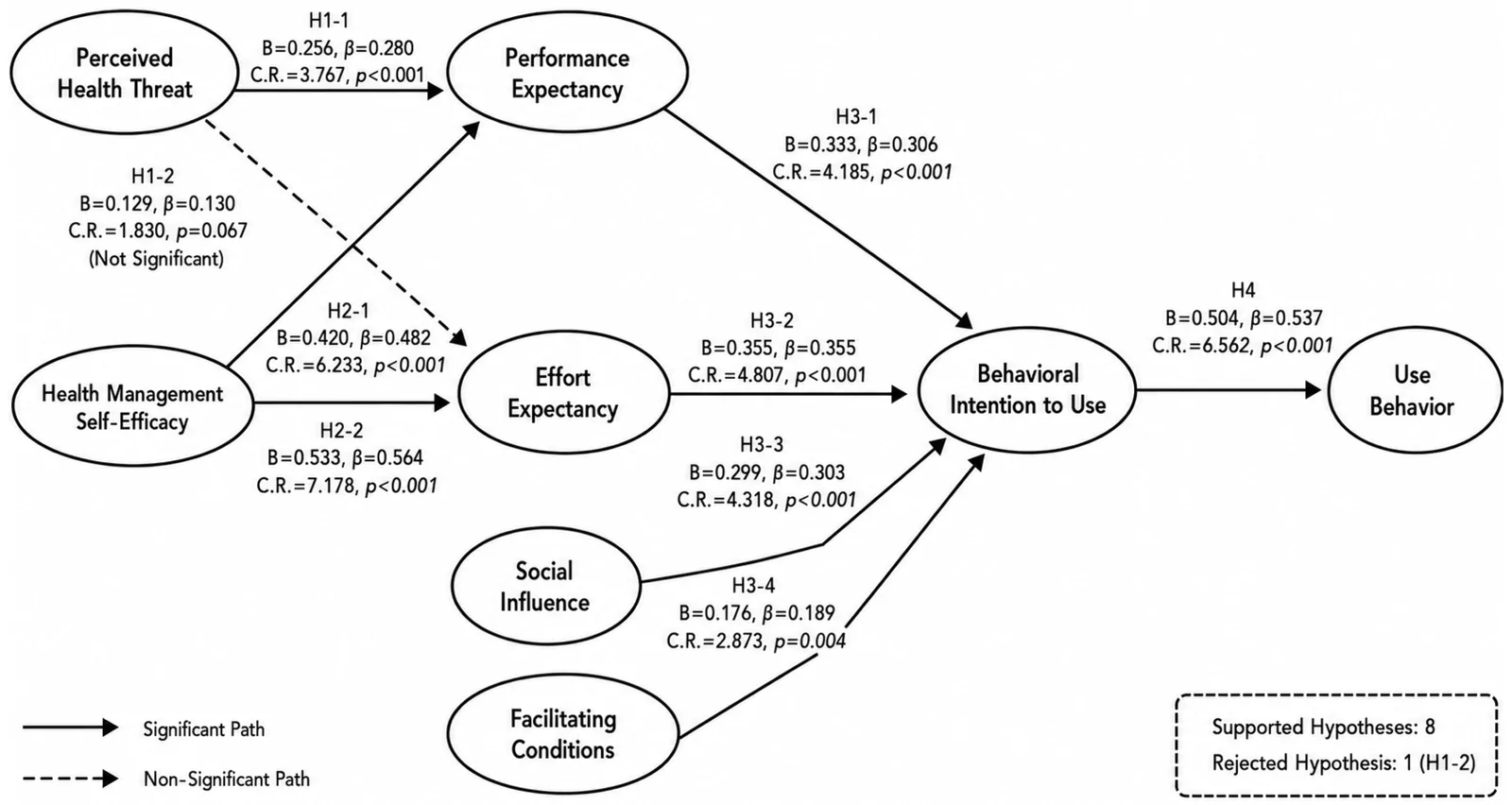
Results of the Structural Model and Hypothesis Testing.

The incremental fit indices indicated a satisfactory model fit, with IFI = 0.982, TLI = 0.980, and CFI = 0.982, all exceeding the recommended criterion of 0.90. Furthermore, the RMSEA was 0.024, with a 90% confidence interval ranging from 0.005 to 0.035, indicating a very low level of approximation error. Although the GFI (0.892), AGFI (0.872), and NFI (0.864) were slightly below the recommended threshold of 0.90, these indices are known to be influenced by sample size and model complexity. Considering all goodness-of-fit indices collectively, the proposed structural model was deemed to provide an adequate representation of the observed data. The overall structural relationships and hypothesis-testing results are also illustrated in Figure 2.

The results of the structural path analysis revealed that eight of the nine hypothesized direct relationships were statistically significant, whereas the path from perceived health threat to effort expectancy was not significant. Perceived health threat exerted a significant positive effect on performance expectancy (β = 0.280, C.R. = 3.767, *p* < 0.001), supporting H1-1. However, its effect on effort expectancy was not statistically significant (β = 0.130, C.R. = 1.830, *p* = 0.067), leading to the rejection of H1-2. Health management self-efficacy had significant positive effects on both performance expectancy (β = 0.482, C.R. = 6.233, *p* < 0.001) and effort expectancy (β = 0.564, C.R. = 7.178, *p* < 0.001), supporting H2-1 and H2-2, respectively. Notably, the effect of health management self-efficacy on effort expectancy was the strongest among the antecedents of effort expectancy. Regarding the UTAUT constructs, performance expectancy positively influenced behavioral intention (β = 0.306, C.R. = 4.185, *p* < 0.001), thereby supporting H3-1. Effort expectancy also had a significant positive effect on behavioral intention (β = 0.355, C.R. = 4.807, *p* < 0.001), representing the strongest predictor of behavioral intention among the four UTAUT antecedents. Likewise, social influence (β = 0.303, C.R. = 4.318, *p* < 0.001) and facilitating conditions (β = 0.189, C.R. = 2.873, *p* = 0.004) positively affected behavioral intention, providing support for H3-3 and H3-4, respectively. Finally, behavioral intention exerted a significant positive effect on use behavior (β = 0.537, C.R. = 6.562, *p* < 0.001), supporting H4. This finding indicates that stronger intentions to use smart healthcare applications are associated with greater actual use behavior among older adults.

### 4.5. Decomposition of Structural Effects

To examine the statistical significance of the indirect effects, a bootstrap analysis with 5000 resamples was conducted, and bias-corrected (BC) 95% confidence intervals (CIs) were estimated as presented in Table 5 [80,81]. The results indicated that perceived health threat exerted a significant indirect effect on behavioral intention through performance expectancy and effort expectancy (B = 0.134, 95% BC CI [0.057, 0.226], *p* = 0.001). Similarly, health management self-efficacy demonstrated a significant indirect effect on behavioral intention (B = 0.329, 95% BC CI [0.235, 0.428], *p* = 0.001).

Furthermore, perceived health threat (B = 0.067, 95% BC CI [0.028, 0.123], *p* = 0.001) and health management self-efficacy (B = 0.166, 95% BC CI [0.108, 0.238], *p* < 0.001) showed significant indirect effects on use behavior through performance expectancy, effort expectancy, and behavioral intention. In addition, performance expectancy, effort expectancy, social influence, and facilitating conditions each exerted significant indirect effects on use behavior through behavioral intention.

Importantly, the bias-corrected 95% confidence intervals for all significant indirect effects did not include zero, providing support for the statistical significance of the mediation effects. These findings indicate that behavioral intention significantly mediates the relationships between the core UTAUT antecedents and actual use behavior among older adults using smart healthcare applications.

### 4.6. Moderation Analysis

To examine the moderating role of technology anxiety, moderation analysis was performed to determine whether technology anxiety moderated the relationships between performance expectancy, effort expectancy, social influence, and behavioral intention. First, mean-centered scores were computed for each independent variable and technology anxiety to reduce potential multicollinearity. Subsequently, interaction terms were created by multiplying each mean-centered independent variable by the corresponding mean-centered technology anxiety score. Hierarchical multiple regression analysis was then conducted with behavioral intention as the dependent variable, including performance expectancy, effort expectancy, social influence, facilitating conditions, technology anxiety, and the interaction terms.

The results of the moderation analysis are presented in Table 6. The interaction effect between performance expectancy and technology anxiety was not statistically significant (β = −0.071, t = −1.083, *p* = 0.280), indicating that technology anxiety did not moderate the relationship between performance expectancy and behavioral intention. Likewise, the interaction effect between effort expectancy and technology anxiety was not significant (β = 0.005, t = 0.087, *p* = 0.931). Therefore, H5-1 and H5-2 were not supported.

In contrast, the interaction effect between social influence and technology anxiety was statistically significant and negative (β = −0.121, t = −2.159, *p* = 0.032), indicating that technology anxiety significantly weakened the positive relationship between social influence and behavioral intention. Accordingly, H5-3 was supported.

To further interpret the significant interaction effect, a simple slope analysis was conducted by estimating the conditional effects of social influence on behavioral intention at three levels of technology anxiety: low (−1 SD), mean, and high (+1 SD), as presented in Table 7.

At the low level of technology anxiety, social influence had a significant positive effect on behavioral intention (B = 0.340, S.E. = 0.072, t = 4.695, *p* < 0.001, 95% CI [0.197, 0.482]). Likewise, the conditional effect remained significant at the mean level of technology anxiety (B = 0.225, S.E. = 0.054, t = 4.201, *p* < 0.001, 95% CI [0.119, 0.331]). In contrast, at the high level of technology anxiety, the effect of social influence on behavioral intention was no longer statistically significant (B = 0.110, S.E. = 0.076, t = 1.446, *p* = 0.150, 95% CI [−0.040, 0.261]). Because the 95% confidence interval included zero, the positive effect of social influence was not supported under conditions of high technology anxiety.

**Figure 3:**
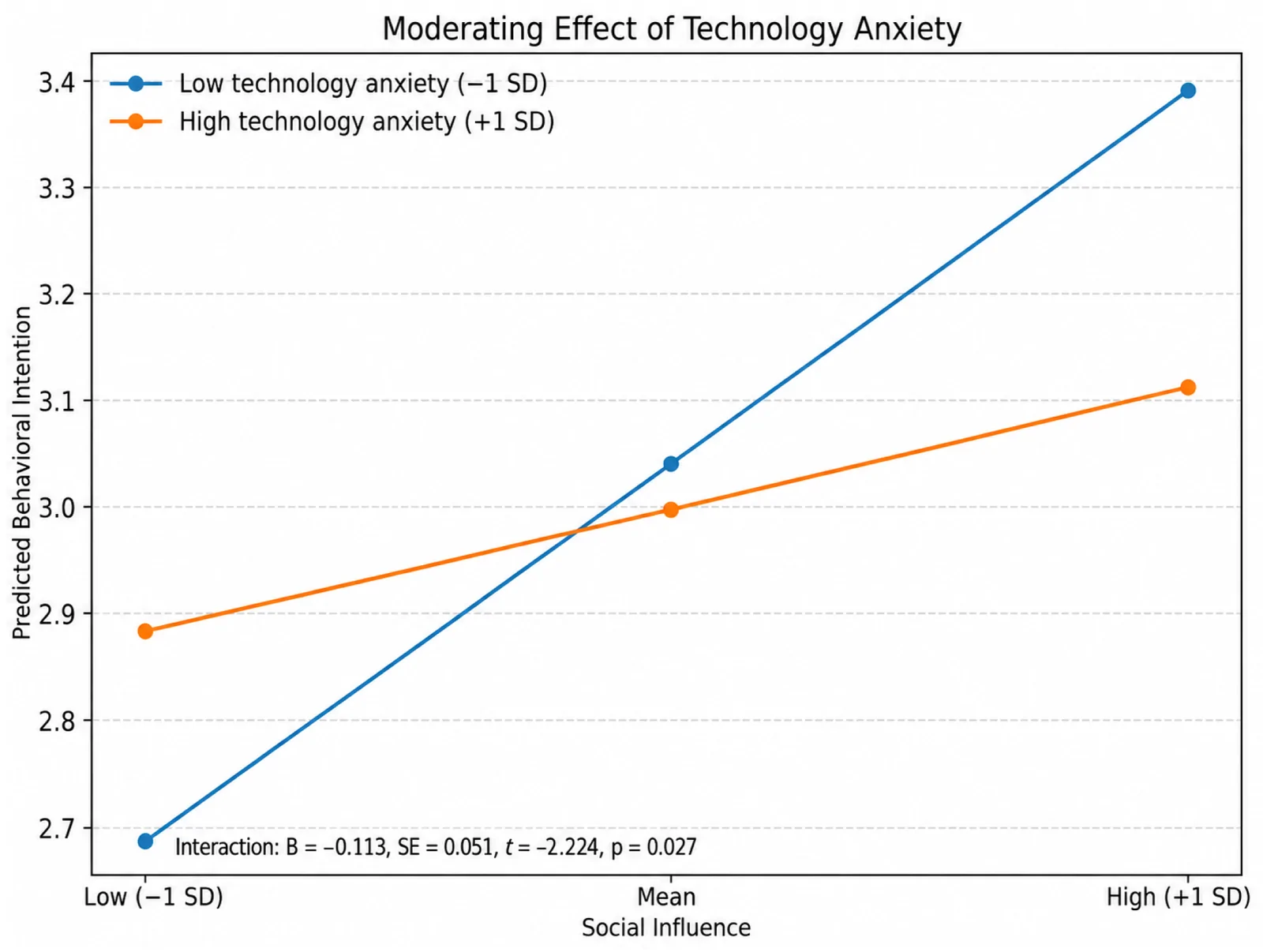
Interaction Plot for the Moderating Effect of Technology Anxiety.

As illustrated in Figure 3, the positive relationship between social influence and behavioral intention became progressively weaker as technology anxiety increased. Specifically, the slope was steepest among older adults with low technology anxiety, whereas it became considerably flatter among those with high technology anxiety, indicating that technology anxiety attenuated the positive influence of social influence on behavioral intention.

## 5. Discussion

This study integrated perceived health threat and health management self-efficacy with UTAUT and examined technology anxiety as a moderating variable to explain older adults’ post-adoption acceptance and continued use of smart healthcare applications. Perceived health threat significantly influenced performance expectancy but not effort expectancy, whereas health management self-efficacy positively influenced both performance expectancy and effort expectancy. Performance expectancy, effort expectancy, social influence, and facilitating conditions significantly explained behavioral intention, which in turn strongly predicted actual use behavior. Thus, the findings clarify the factors underlying continued engagement with smart healthcare applications among older adults with prior use experience.

First, perceived health threat was significantly and positively associated with performance expectancy (β = 0.280, *p* < 0.001) but was not significantly associated with effort expectancy (β = 0.130, *p* = 0.067). This contrast suggests that greater perceived health risk strengthens older adults’ expectations regarding the health-management benefits of smart healthcare applications without necessarily making the technology appear easier to use. This finding is consistent with the Health Belief Model, which emphasizes perceived health threats as motivators of preventive behavior [38], and with previous research on mobile healthcare acceptance [15]. The nonsignificant association with effort expectancy further suggests that perceived need for healthcare and perceived ease of technology use represent distinct psychological evaluations. Thus, health concerns may increase the perceived value of smart healthcare applications, whereas ease-of-use perceptions may depend more on digital experience and interface characteristics.

Second, health management self-efficacy was significantly and positively associated with both performance expectancy (β = 0.482, *p* < 0.001) and effort expectancy (β = 0.564, *p* < 0.001). The stronger association with effort expectancy suggests that confidence in managing one’s health is particularly relevant to perceptions that smart healthcare applications can be learned and used with relatively little difficulty. This finding supports Bandura’s [47] self-efficacy perspective and previous studies emphasizing self-efficacy in digital healthcare adoption [14,82]. The results therefore suggest that health management self-efficacy is not limited to motivating health behavior but also shapes how older adults evaluate the usability and practical value of digital health technologies.

Third, all four UTAUT constructs were significantly and positively associated with behavioral intention: performance expectancy (β = 0.306, *p* < 0.001), effort expectancy (β = 0.355, *p* < 0.001), social influence (β = 0.303, *p* < 0.001), and facilitating conditions (β = 0.189, *p* = 0.004). Among these relationships, effort expectancy showed the strongest association, although its magnitude was relatively close to those of performance expectancy and social influence. This pattern supports the UTAUT framework [11] while highlighting the importance of usability in smart healthcare adoption among older adults. The findings suggest that adoption is shaped not only by expected health benefits but also by whether the application appears manageable and whether its use is supported by significant others and an adequate support environment. This interpretation is consistent with previous research emphasizing the importance of usability and social and environmental support in older adults’ technology adoption [8,61,62].

Fourth, behavioral intention was strongly and positively associated with actual use behavior (β = 0.537, *p* < 0.001), representing the largest standardized coefficient among the relationships examined for technology adoption outcomes. This finding confirms the central proposition of UTAUT that behavioral intention is a proximal determinant of actual technology use and is also consistent with the Theory of Planned Behavior [83]. Moreover, the significant indirect effects of performance expectancy, effort expectancy, social influence, and facilitating conditions on use behavior through behavioral intention further support this mechanism. Thus, the findings suggest that positive evaluations of smart healthcare applications and supportive social and technical conditions are associated with actual use through behavioral intention.

Finally, technology anxiety exhibited a selective moderating effect on the technology acceptance process. The interaction between technology anxiety and performance expectancy was not significant (β = −0.071, *p* = 0.280), nor was the interaction between technology anxiety and effort expectancy (β = 0.005, *p* = 0.931). In contrast, technology anxiety significantly weakened the positive relationship between social influence and behavioral intention (β = −0.121, *p* = 0.032). This pattern indicates that technology anxiety does not uniformly undermine the effects of older adults’ own evaluations of usefulness or ease of use; rather, it primarily constrains the extent to which external social encouragement is translated into behavioral intention. The conditional effects further clarify this moderating mechanism. When technology anxiety was low (−1 SD), social influence had a strong positive effect on behavioral intention (B = 0.340, *p* < 0.001), and the effect remained significant at the mean level of technology anxiety (B = 0.225, *p* < 0.001). However, when technology anxiety was high (+1 SD), the effect decreased substantially and was no longer statistically significant (B = 0.110, *p* = 0.150). Thus, recommendations from family members, healthcare professionals, or other significant individuals appear to be effective primarily when older adults experience relatively low or moderate levels of technology anxiety. Importantly, these findings suggest that simply encouraging older adults with high technology anxiety to use smart healthcare applications may have limited effectiveness. Repeated recommendations without addressing underlying anxiety may be perceived as pressure and could potentially reinforce reluctance or resistance toward technology use. Therefore, social encouragement should be accompanied by measures that reduce technology anxiety, such as hands-on assistance, simplified interfaces, and gradual learning experiences that build confidence before independent use. In this sense, reducing technology anxiety may be an important prerequisite for social influence to effectively facilitate technology adoption among older adults.

The selective nature of this moderating effect also extends the UTAUT perspective. While social influence is generally assumed to promote behavioral intention [11], the present findings demonstrate that its effectiveness may depend on an individual’s psychological readiness to engage with technology. Technology anxiety can therefore be understood as a psychological boundary condition that determines when social influence facilitates smart healthcare application adoption among older adults.

## 6. Implications and Conclusions

This study provides an integrated theoretical explanation of older adults’ smart healthcare application use by linking perceived health threat and health management self-efficacy from HBM to the core UTAUT process and positioning technology anxiety as a psychological boundary condition. Rather than examining health beliefs, technology acceptance factors, and technology anxiety separately, the proposed framework explains how health-related beliefs are associated with technology expectations, how these expectations are related to behavioral intention and actual use behavior, and when these relationships vary according to technology anxiety. The findings showed that perceived health threat was associated with performance expectancy but not effort expectancy, whereas health management self-efficacy was positively associated with both. All four UTAUT constructs were significantly associated with behavioral intention, which showed the strongest association with actual use behavior. Technology anxiety selectively moderated the relationship between social influence and behavioral intention, demonstrating the value of integrating HBM, UTAUT, and technology anxiety within a single framework for explaining older adults’ smart healthcare use.

This study provides several theoretical implications. First, integrating HBM and UTAUT demonstrates how health-related motivations and technology acceptance mechanisms jointly explain smart healthcare use. Perceived health threat and health management self-efficacy shape performance and effort expectancy, which subsequently contribute to behavioral intention and use behavior. This integration extends technology acceptance research by positioning health beliefs as antecedents of technology evaluations. Second, health management self-efficacy positively influenced both performance expectancy and effort expectancy, with a stronger effect on the latter. This suggests that older adults’ confidence in managing their health is particularly important in shaping perceived ease of use. Thus, self-efficacy represents not only a determinant of health behavior but also an important psychological factor in continued digital health technology use. Third, this study identifies the selective moderating role of technology anxiety. Technology anxiety did not significantly moderate the effects of performance expectancy or effort expectancy on behavioral intention but significantly weakened the effect of social influence. This finding suggests that technology anxiety functions as a psychological boundary condition that limits the effectiveness of social encouragement rather than uniformly affecting the technology acceptance process, thereby extending the explanatory scope of UTAUT among older adults.

The findings also provide several practical implications. First, developers of smart healthcare applications should prioritize intuitive user interfaces, simplified navigation, and user-friendly interaction rather than merely increasing functional complexity. Given that effort expectancy was the strongest predictor of behavioral intention, improving ease of use is likely to be more effective than expanding application functionality. Second, educational programs designed to enhance older adults’ health management self-efficacy through hands-on training and repeated practice may facilitate greater acceptance and continued use of smart healthcare applications. Third, establishing supportive ecosystems involving family members, healthcare professionals, and local communities is essential for promoting technology adoption among older adults. However, because technology anxiety substantially weakens the positive influence of social support, social encouragement alone is insufficient for older adults with high levels of technology anxiety. Accordingly, practical support strategies—including digital literacy education, initial installation assistance, voice-guided interfaces, and AI-based user support services—should be implemented to reduce technology anxiety and facilitate technology adoption.

Overall, this study extends technology acceptance research by integrating health-related beliefs with UTAUT and identifying the moderating role of technology anxiety in explaining post-adoption acceptance and continued use of smart healthcare applications among older adults. By examining health motivation, self-efficacy, technology acceptance factors, and technology anxiety within an integrated framework, the study provides a more context-sensitive explanation of continued smart healthcare application use. The findings provide practical evidence for designing digital healthcare services that support sustained engagement among older adults and offer a foundation for future research on post-adoption digital healthcare behavior.

## 7. Limitations and Future Research

This study has several limitations that should be acknowledged. First, because the study employed a cross-sectional design and self-reported survey data, causal relationships should be interpreted with caution. In addition, use behavior was measured entirely through self-reports, without objective indicators such as usage frequency, duration, application logs, or system-generated data. Future studies should employ longitudinal designs and incorporate objective usage data to more accurately assess continued technology use over time. Second, participants were recruited through the relatives and acquaintances of university students, constituting a convenience sample that may be subject to selection bias. In addition, the sample was limited to South Korean adults aged 60 years and above with prior experience using smart healthcare applications. Therefore, caution is required when generalizing the findings. Future studies should employ more representative sampling methods and include diverse populations and cultural contexts. Finally, although this study examined technology anxiety as a moderating variable, future research could extend the proposed framework by incorporating additional psychological and contextual factors, such as digital literacy, trust, privacy concerns, and eHealth literacy. Including these variables would provide a more comprehensive understanding of the mechanisms underlying older adults’ adoption of smart healthcare technologies.

## Figures and Tables

**Table 1:** Demographic Characteristics of the Respondents.

Category	Item	Frequency (*n*)	Percentage (%)
Gender	Male	98	43.8
	Female	126	56.3
Age	60–64 years	101	45.1
	65–69 years	79	35.3
	70 years or older	44	19.6
Self-rated Health Status	Excellent	3	1.3
	Good	37	16.5
	Fair	99	44.2
	Poor	75	33.5
	Very poor	10	4.5
Duration of App Use	Less than 6 months	49	21.9
	6 months to <1 year	54	24.1
	1–3 years	62	27.7
	More than 3 years	59	26.3
Frequency of App Use	More than three times per day	16	7.1
	Once or twice per day	53	23.7
	Two to four times per week	103	46.0
	One to three times per month	52	23.2
Primary Type of App Used	Fitness & Activity	69	30.8
	Diet & Nutrition	58	25.9
	Medical & Chronic Disease Management	26	11.6
	Sleep & Mental Health	32	14.3
	Medical Information & Telehealth	18	8.0
	Community & Gamification	21	9.4
Total		224	100.0

**Table 2:** Measurement Model Reliability and Convergent Validity.

Construct	Measurement Item	Standardized Factor Loading	Cronbach’s α	CR	AVE
Perceived Health Threat	PHT1	0.754	0.825	0.826	0.543
	PHT2	0.715			
	PHT3	0.763			
	PHT4	0.715			
Health Management Self-Efficacy	HMSE1	0.836	0.848	0.850	0.587
	HMSE2	0.734			
	HMSE3	0.771			
	HMSE4	0.718			
Performance Expectancy	PE1	0.762	0.826	0.828	0.548
	PE2	0.795			
	PE3	0.740			
	PE4	0.657			
Effort Expectancy	EE1	0.778	0.838	0.839	0.567
	EE2	0.760			
	EE3	0.762			
	EE4	0.709			
Social Influence	SI1	0.798	0.792	0.792	0.559
	SI2	0.720			
	SI3	0.722			
Facilitating Conditions	FC1	0.832	0.843	0.845	0.579
	FC2	0.752			
	FC3	0.675			
	FC4	0.776			
Technology Anxiety	TA1	0.764	0.828	0.828	0.548
	TA2	0.768			
	TA3	0.754			
	TA4	0.669			
Behavioral Intention	BI1	0.835	0.837	0.839	0.635
	BI2	0.752			
	BI3	0.801			
Use Behavior	UB1	0.794	0.790	0.792	0.559
	UB2	0.753			
	UB3	0.693			

Note. CR = Composite Reliability; AVE = Average Variance Extracted. All standardized factor loadings were statistically significant at *p* < 0.001.

**Table 3:** Correlations and Discriminant Validity of the Constructs.

Variables	PHT	HMSE	PE	EE	SI	FC	TA	BI	UB
PHT	0.737								
HMSE	0.133 *	0.766							
PE	0.302 **	0.457 **	0.740						
EE	0.179 **	0.490 **	0.277 **	0.753					
SI	0.097	0.019	−0.022	0.115	0.748				
FC	0.012	0.184 **	0.082	0.311 **	0.117	0.761			
TA	0.066	−0.119	−0.098	−0.148 *	0.002	−0.157 *	0.740		
BI	0.226 **	0.311 **	0.359 **	0.470 **	0.284 **	0.314 **	−0.118	0.797	
UB	0.193 **	0.260 **	0.223 **	0.262 **	0.207 **	0.201 **	−0.086	0.418 **	0.748

Note: PHT = Perceived Health Threat; HMSE = Health Management Self-Efficacy; PE = Performance Expectancy; EE = Effort Expectancy; SI = Social Influence; FC = Facilitating Conditions; TA = Technology Anxiety; BI = Behavioral Intention; UB = Use Behavior. Diagonal values in bold represent the square root of the average variance extracted (AVE); off-diagonal values represent Pearson correlation coefficients. * *p* < 0.05; ** *p* < 0.01.

**Table 4:** Structural Path Analysis and Hypothesis Testing.

Hypothesis	Structural Path	B	S.E.	β	C.R.	*p*	Result
H1-1	PHT → PE	0.256	0.068	0.280	3.767	<0.001	Supported
H1-2	PHT → EE	0.129	0.070	0.130	1.830	0.067	Rejected
H2-1	HMSE → PE	0.420	0.067	0.482	6.233	<0.001	Supported
H2-2	HMSE → EE	0.533	0.074	0.564	7.178	<0.001	Supported
H3-1	PE → BI	0.333	0.079	0.306	4.185	<0.001	Supported
H3-2	EE → BI	0.355	0.074	0.355	4.807	<0.001	Supported
H3-3	SI → BI	0.299	0.069	0.303	4.318	<0.001	Supported
H3-4	FC → BI	0.176	0.061	0.189	2.873	0.004	Supported
H4	BI → UB	0.504	0.077	0.537	6.562	<0.001	Supported
Model fit: χ^2^ = 415.052, df = 367, χ^2^/df = 1.131, CFI = 0.982, TLI = 0.980, IFI = 0.982, RMSEA = 0.024 (90% CI [0.005, 0.035]), PCLOSE = 1.000.							

Note. *B* = Unstandardized path coefficient; *S.E.* = Standard error; *β* = Standardized path coefficient; *C.R.* = Critical ratio.

**Table 5:** Direct, Indirect, and Total Effects.

Predictor	Outcome	Direct Effect	Indirect Effect	Total Effect	Bootstrap *p*	Bias-Corrected 95% CI	Result
PHT	PE	0.258	—	0.258	<0.001	[0.118, 0.408]	Significant
PHT	EE	0.130	—	0.130	0.070	[−0.009, 0.279]	Not significant
HMSE	PE	0.418	—	0.418	<0.001	[0.297, 0.552]	Significant
HMSE	EE	0.524	—	0.524	<0.001	[0.393, 0.670]	Significant
PHT	BI	—	0.134	0.134	0.001	[0.057, 0.226]	Significant
HMSE	BI	—	0.329	0.329	0.001	[0.235, 0.428]	Significant
PE	BI	0.338	—	0.338	<0.001	[0.176, 0.515]	Significant
EE	BI	0.357	—	0.357	0.001	[0.208, 0.509]	Significant
SI	BI	0.300	—	0.300	<0.001	[0.181, 0.439]	Significant
FC	BI	0.185	—	0.185	0.004	[0.058, 0.323]	Significant
PHT	UB	—	0.067	0.067	0.001	[0.028, 0.123]	Significant
HMSE	UB	—	0.166	0.166	<0.001	[0.108, 0.238]	Significant
PE	UB	—	0.170	0.170	<0.001	[0.085, 0.283]	Significant
EE	UB	—	0.180	0.180	<0.001	[0.100, 0.276]	Significant
SI	UB	—	0.151	0.151	<0.001	[0.084, 0.242]	Significant
FC	UB	—	0.093	0.093	0.003	[0.029, 0.177]	Significant
BI	UB	0.504	—	0.504	<0.001	[0.367, 0.658]	Significant

Note. Effect estimates are based on the unstandardized effects obtained from the AMOS output. Bootstrap significance was evaluated using 5000 bias-corrected bootstrap resamples. CI= Bias-corrected confidence interval.

**Table 6:** Moderating Effect of Technology Anxiety.

Hypothesis	Interaction Path	β	t	*p*	Result
H5-1	Performance Expectancy × Technology Anxiety → Behavioral Intention	−0.071	−1.083	0.280	Rejected
H5-2	Effort Expectancy × Technology Anxiety → Behavioral Intention	0.005	0.087	0.931	Rejected
H5-3	Social Influence × Technology Anxiety → Behavioral Intention	−0.121	−2.159	0.032	Supported

**Table 7:** Conditional Effect of Social Influence on Behavioral Intention Across Levels of Technology Anxiety.

Level of Technology Anxiety	Effect (B)	S.E.	t	*p*	LLCI	ULCI	Interpretation
Low (−1 SD)	0.340	0.072	4.695	<0.001	0.197	0.482	Significant
Mean	0.225	0.054	4.201	<0.001	0.119	0.331	Significant
High (+1 SD)	0.110	0.076	1.446	0.150	−0.040	0.261	Not Significant

## Data Availability

The data presented in this study are available on request from the corresponding author due to privacy considerations related to the survey participants.
